# The Intervention Effect of Externally Applying or Orally Administering *Bifidobacterium animalis* Subsp. *lactis* J12 on Atopic Dermatitis Induced by 2,4-Dinitrofluorobenzene

**DOI:** 10.3390/microorganisms14020274

**Published:** 2026-01-24

**Authors:** Yan Zhang, Yixuan Jiang, Weilian Hong, Shaoyang Ge, Nanqing Jing, Jianjun Yang, Yuanhong Xie, Hongxing Zhang, Junhua Jin

**Affiliations:** 1 College of Food Science and Engineering, Beijing University of Agriculture, Beijing 102206, China; zyan19970727@163.com (Y.Z.); 202430611007@bua.edu.cn (Y.J.); geshaoyang@foxmail.com (S.G.); 17331287068@163.com (N.J.); 20107803@bua.edu.cn (Y.X.); 20057001@bua.edu.cn (H.Z.); 2 National Center of Technology Innovation for Dairy, Hohhot 010080, China; 13774299154@163.com; 3 Laboratory of Precision Nutrition and Food Quality, Minstry of Education, Department of Nutrition and Health, China Agricultural University, Beijing 100083, China; 18811626398@163.com

**Keywords:** atopic dermatitis, *Bifidobacterium animalis*, immune response, gut microbiota, skin barrier

## Abstract

Atopic dermatitis (AD) is a chronic, relapsing inflammatory skin disease with critical unmet therapeutic needs. This study compared the efficacy of three probiotics—*Bifidobacterium animalis* subsp. *lactis* J12 (*B. animalis* J12), *Lactiplantibacillus plantarum* Zhang-LL (*L. plantarum* Zhang-LL), and *Limosilactobacillus salivarius* M18-6 (*L. salivarius* M18-6)—in a 2,4-dinitrofluorobenzene (DNFB)-induced mouse AD model. Interventions included topical fermented supernatants (J12S/Z-LL-S/M18-6-S), oral live cells (J12L/Z-LL-L/M18-6-L), and topical dexamethasone (Dex) as the positive control. Post-intervention, AD-related pathological and immunological indices were evaluated. Among the three probiotics, J12 exhibited superior efficacy, whereas *L. plantarum* Zhang-LL and *L. salivarius* M18-6 showed limited therapeutic effects. Both J12-derived formulations alleviated DNFB-induced AD symptoms: Topical J12S significantly reduced ear swelling, serum IL-4 and IL-17 levels, and increased the proportion of splenic Treg cells. Oral J12L exerted comparable immunomodulatory effects, while further improving skin pathology—epidermal thickness and mast cell infiltration were each reduced to approximately one-third of those in the model group. Additionally, J12L regulated gut microbiota by enhancing alpha diversity and altering functional predictions. Collectively, *B. animalis* J12 is a promising candidate for AD management: topical J12S serves as an effective, non-invasive alternative to oral J12L. Notably, the two formulations act through distinct yet complementary mechanisms—J12L exerts systemic effects via regulating immunity and the gut–skin axis, while J12S exerts local anti-inflammatory effects and protects the skin barrier—highlighting J12′s versatile therapeutic potential for AD.

## 1. Introduction

Atopic dermatitis (AD) is a chronic inflammatory skin disease characterized by pruritus (itching). It is considered a special type of skin inflammation. The incidence of AD is approximately 15–20% in children and 2–10% in adults [1,2]. The prevalence of AD has been steadily increasing over the past few decades, not only in urban and economically developed countries but also in developing nations. The primary symptoms of AD include damage to the stratum corneum (outermost layer of the skin) and increased itching [3,4]. Damage to the skin barrier is the initial step in the development of AD [5]. When external factors impact the body’s immune system, it triggers an immune response and leads to the development of AD. This immune dysregulation is characterized by a pronounced imbalance in T helper (Th) cell responses, specifically a shift towards a type 2 (Th2)-dominant immunity. Th2 cells drive allergic inflammation through cytokines such as interleukin-4 (IL-4), IL-5, and IL-13, which promote IgE production, eosinophil activation, and skin barrier dysfunction [6]. The gut microbiota acts as a critical mediator in the “gut–skin” axis, influencing the physiological function of the skin system and the pathological processes of associated skin diseases by regulating metabolism, immunity, and endocrine functions [7,8].

Studies have demonstrated that the oral administration of *Bifidobacterium longum* or *Bifidobacterium brevis* live cells can improve skin aging induced by ultraviolet rays (UVB), reducing the formation of skin wrinkles and mitigating oxidative stress and collagen degradation during the aging process [9,10,11]. Additionally, oral administration of *B. longum* live cells can ameliorate symptoms of AD induced by 2,4-dinitrofluorobenzene (DNFB) and restore skin barrier function [12]. Furthermore, *Lactobacillus plantarum* LP22A3 has been shown to influence allergenic mast cells through intestinal epithelial cells [13]. Oral administration of *L. plantarum* IS-10506 microcapsules has been found to reduce the severity scoring of atopic dermatitis (SCORAD) in children with AD [14]. Oral administration of *L. plantarum* NCIMB8826 live cells has demonstrated the ability to decrease colon mast cell counts and improve skin pathology. It achieves this by enhancing skin barrier integrity, regulating intestinal immune homeostasis, and ultimately aiding in the suppression of AD. Additionally, oral *L. salivarius* LA307 lyophilized powder has been found to maintain skin integrity and homeostasis by regulating the intestinal flora [15]. Oral *L. salivarius* LS01 at a dose of 10^9^ colony-forming units CFU/g in maltodextrin has demonstrated efficacy in reducing the SCORAD score and itch index in patients with adult AD. It also plays an important role in regulating T helper type 1 (Th1) and T helper type 2 (Th2) cell cytokines and is considered an important adjunct therapy for AD treatment [16]. However, there are currently no reports of using probiotic fermentation supernatant to smear to the skin for the treatment of AD.

*Bifidobacterium* stands out as one of the most extensively studied probiotic species, thoroughly researched across various aspects of human health. Notably, it plays a crucial role in promoting skin health, and is predominantly present in the intestines. Our research group has previously conducted investigations into the impact of *B. animalis* J12 on multiple aspects of health. In a previous study, we discovered that *B. animalis* J12 can prevent hyperglycemia during pregnancy and improve the pathology and nervous system of rat offspring. This effect is achieved through the regulation of placental flora, ultimately influencing the health of the future generations [17]. Additionally, *B. animalis* J12 has been shown to decrease the levels of Interleukin-1 beta (IL-1β), glutathione and malondialdehyde in the serum of oral ulcer golden hamsters. It also reduces the expression of nuclear factor κB (NF-κB) and matrix metalloproteinases (MMPs), key drivers of inflammation and tissue degradation, thereby lowering overall inflammation levels and DNA damage in ulcer tissue. These interventions contribute to the promotion of oral mucosal health [18]. Beyond Bifidobacterium, other probiotic strains with specific regulatory capabilities have also garnered attention for their potential in health promotion. Specifically, *Lactiplantibacillus plantarum* Zhang-LL (*L. plantarum* Zhang-LL) has been demonstrated to possess significant immune system regulatory ability, while *Limosilactobacillus salivarius* M18-6 (*L. salivarius* M18-6) exhibits prominent antioxidant capabilities [19,20]. These functional properties are closely associated with the regulation of inflammatory responses, a process that has been well-elucidated in fundamental research [21,22]. Given their respective functional advantages, these two strains were selected for comparative analysis in the present study. Therefore, this study aimed to comparatively evaluate the therapeutic effects of *B. animalis* J12 (J12) on DNFB-induced atopic dermatitis in mice via two distinct application routes (oral live cells and topical fermented supernatant), preliminarily compare J12′s efficacy with the aforementioned two probiotic strains, and explore the underlying mechanisms, including systemic and skin-localized immune modulation, skin barrier restoration, and alterations in gut microbiota composition and function.

## 2. Materials and Methods

### 2.1. Microbiology Experiment

*B. animalis* J12 (CGMCC No.25005) were isolated from the feces of healthy infants. *B. animalis* J12 was inoculated in Man–Rogosa–Sharpe (MRS) liquid medium with 0.05% cysteine hydrochloride for amplification and cultured to the third generation in an anaerobic culture environment. *L. plantarum* Zhang-LL (CGMCC No.6936) and *L. salivarius* M18-6 (CGMCC No.16199) were cultured in the MRS in an aerobic culture environment. After counting, the cell suspension concentration was diluted to 5.0 × 10^9^ CFU/mL with normal saline. The fermentation supernatant was filtered through a 0.22 μm filter membrane for later use.

### 2.2. Animal Experimental Design

**Figure 1:**
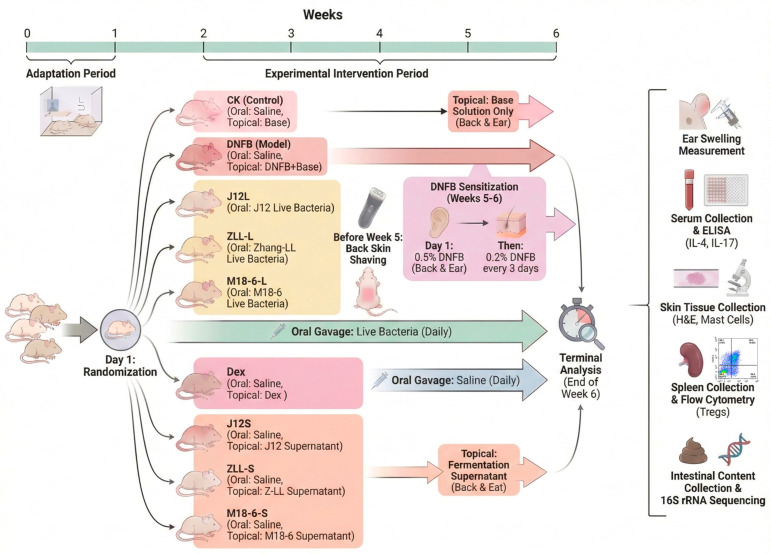
The schedule of the experimental protocol and drug administration (*n* = 12 per group).

All animal experiments complied with the Animal Management Regulations of the Ministry of Science and Technology of the People’s Republic of China and were approved by the Institutional Review Board (or Ethics Committee) of Beijing Gushi Test Technology Co., Ltd., Beijing, China, Laboratory Animal Welfare and Animal Experimental Ethical Committee (protocol code GSCS-2025-007, 29 December 2024); female 6-week-old BALB/c mice purchased from Beijing Weitong Lihua Experimental Animal Technology Co., Ltd., Beijing, China, were given a one-week adaptation period in a controlled environment (temperature: 22 ± 2 °C, relative humidity: 50 ± 10%, 12 h light/dark cycle) upon arrival, with free access to food and water to acclimate to the experimental conditions. A double-blinding strategy was implemented throughout the experiment (researchers responsible for grouping, reagent preparation, intervention, and endpoint evaluation were blinded to group assignments, with group codes decoded post data collection and preliminary statistical analysis), and after adaptation, mice were randomized into 9 groups (12 mice per group, *n* = 12) using a random number table method with GraphPad Prism 9 software to minimize baseline differences in body weight and skin condition, where the sample size was doubled from the 6 mice per group used in previous DNFB-induced AD model studies [8] to enhance statistical power for multiple detection endpoints (e.g., clinical AD scoring, histopathological analysis, serum cytokine quantification, gut microbiota profiling), improve result stability and reproducibility by reducing the impact of outliers from individual variability in probiotic interventions, and account for potential attrition (e.g., accidental death, invalid samples) during the experiment, with the final number of valid samples per group 7–10 meeting statistical validity requirements. The 9 groups included the CK group (blank control, fed with normal saline), DNFB group (AD model control, applied with 2,4-dinitrofluorobenzene), positive control group (treated with topical dexamethasone, Dex), J12L group (fed with live cells of *B. animalis* J12), ZLL-L group (fed with live cells of *L. plantarum* Zhang-LL), M18-6-L group (fed with live cells of *L. salivarius* M18-6), J12S group (applied with fermentation supernatant of *B. animalis* J12), ZLL-S group (applied with fermentation supernatant of *L. plantarum* Zhang-LL), and M18-6-S group (applied with fermentation supernatant of *L. salivarius* M18-6); the vehicles used were sterile phosphate-buffered saline (PBS) for oral gavage of live probiotics and a 4:1 (*v*/*v*) mixture of acetone and olive oil as the base solution for DNFB, with sterility (no bacterial/fungal growth on LB/Sabouraud agar plates after 24–48 h incubation at 37 °C) and biocompatibility (no abnormalities in a subset of mice, *n* = 3, administered with vehicles) verified to ensure safety and non-interference with experimental outcomes. Live probiotic suspensions were prepared by resuspending the strains in sterile PBS (washed three times) and were freshly made daily before use; starting from the second week, mice in the J12L/ZLL-L/M18-6-L groups were orally administered the corresponding live probiotic suspensions, while those in the other groups received an equivalent volume of sterile saline via oral gavage. AD model establishment was initiated in the fifth week: prior to modeling, the back skin and hair of mice were shaved to a 2.5 cm × 1.5 cm area, 0.5% and 0.2% DNFB solutions were prepared with the acetone–olive oil base solution, 50 µL of 0.5% DNFB solution was applied to the shaved back for sensitization on the first day of modeling, and thereafter 50 µL of 0.2% DNFB solution was applied to the back skin and 10 µL of 0.2% DNFB to the right ear every three days to induce and maintain AD symptoms, with the CK group receiving the same amount of base solution without DNFB, and the J12S/ZLL-S/M18-6-S groups topically treated with 200 µL of 0.22 µm filtered fermentation supernatant starting from modeling initiation; the experiment lasted for six weeks. The experimental animal scheme is shown in Figure 1.

### 2.3. Measurement of Ear Swelling in Mice

At the end of the experiment, the thickness of the left and right ears of the mice was measured, and the ear swelling rate was calculated. Ear thickness changes = right ear thickness-left ear thickness.

### 2.4. Measurement of Serum Inflammatory Factors

Blood was coagulated at 20 °C for one hour and then centrifuged at 12,000 rpm and 4 °C for 10 min. IL-4 and IL-17 were measured according to the instructions of the kit.

### 2.5. Measurement of Skin Inflammatory Factors

The skin tissue samples were quickly ground with liquid nitrogen, and the ground skin samples (100 mg) were placed in 500 μL RIPA lysate containing 2% (*v*/*v*) protease inhibitor mixture and 2% (*v*/*v*) phosphatase inhibitor mixture, and lysed on ice. After lysis, the supernatant was centrifuged. Total protein content was measured using a bicinchoninic acid (BCA) assay, and IL-4, IL-17 levels were measured using a specific enzyme-linked immunosorbent assay (ELISA).

### 2.6. Histological Examination

Mouse skin was preserved in 4% paraformaldehyde solution, embedded in paraffin, sectioned, and stained with hematoxylin and eosin. Micrographs were scanned using a digital slide scanner, and symptoms of slide pathology were evaluated by a professional. Then, 5 µm thick sections were baked, dehydrated, stained with mast cell staining solution (toluidine blue), differentiated, made transparent, sealed and made into pathological sections. Mast cells were counted under a light microscope in three different fields.

### 2.7. Flow Cytometric Analysis of Splenic Regulatory T (Treg) Cells

The population of splenic regulatory T cells was analyzed by flow cytometry. Briefly, mice were euthanized and spleens were aseptically removed. After trimming away adherent connective tissue, each spleen was placed in a 5 mL centrifuge tube containing 2 mL of ice-cold phosphate-buffered saline (PBS) and mechanically dissociated using surgical scissors. The spleen was passed through a 200 mesh nylon screen and centrifuged at 300 *g* at 4 °C for 5 min, and the supernatant was poured. Then, 1× red blood cell lysate was added and lysed for 4 min. It was passed through a 200 mesh nylon screen again; the samples were centrifuged at 300 *g* for 5 min at 4 °C, and the supernatant was discarded. The cells were resuspended with PBS, and the concentration of cells was between 10^5^ and 10^8^. The resuspended cells were aliquoted into four 100 μL portions in flow cytometry tubes. These tubes were designated as follows: the unstained control (NC), the CD4 single-stained control, the Foxp3 single-stained control, and the Treg staining sample (stained for CD4, CD25, and Foxp3). CD4 tubes had 0.125 μg CD4 antibody added, and Treg tubes had 0.125 μg CD4 antibody and 0.06 μg CD25 antibody added, respectively. Then, CD4 and Treg tubes were placed in a dark place for 20 min, and NC and Foxp3 tubes were placed on ice until use. After 20 min in the dark, the samples were washed with 1 mL flow cytometry staining buffer, centrifuged at 300 *g* for 5 min at 4 °C, and the supernatant was discarded. CD4 tubes were resuspended in 400 μL flow cytometry staining buffer, and then cells were stained with NC, Foxp3 and Treg cells. NC, Foxp3 and Treg tubes with 1 mL 1X fixed liquid membrane were kept in a dark place for 30 to 60 min, avoiding light, and 2 mL 1X permeabilization buffer added after, 300 *g* 4 °C centrifuge for 5 min, and then the supernatant was abandoned. For the Foxp3 tube and Treg tube, respectively, 100 μL 1X Permeabilization Buffer was added, and refrigerated at 4 °C. For the Foxp3 antibody, respectively, 0.5 μg Foxp3 antibody was added to the Foxp3 tube and Treg tube, and light avoided for 1 h. The NC tube had 400 μL flow cytometry staining buffer added before waiting for loading. The samples were centrifuged at 300 *g* for 5 min at 4 °C and the supernatant was discarded. The Foxp3 tube and Treg tube were resuspended in 400 μL flow cytometry staining buffer before waiting for the machine.

### 2.8. Detection of the Gut Microbial Diversity

Sample Collection: At the experimental endpoint, colonic luminal contents were aseptically collected and immediately frozen at −80 °C. Microbial genomic DNA was extracted from intestinal contents. The quality and concentration of the extracted DNA were assessed by 1% agarose gel electrophoresis. The V3–V4 hypervariable region of the bacterial 16S rRNA gene was amplified by polymerase chain reaction (PCR). The resulting PCR products were purified using the AxyPrep DNA Gel Extraction Kit (Axygen Biosciences, Union City, CA, USA), quantified fluorometrically, and used to construct sequencing libraries. Paired-end sequencing (2 × 250 bp) was performed on an Illumina NovaSeq platform (Illumina, San Diego, CA, USA). Bioinformatics analysis was performed on the Majorbio Cloud Platform (https://cloud.majorbio.com).

### 2.9. Bioinformatics and Biostatistical Analysis of 16S rRNA Sequencing Data

Microbiome bioinformatics and statistical analyses were primarily performed using the Majorbio Cloud Platform (https://cloud.majorbio.com). Alpha and Beta Diversity Analysis: Alpha diversity indices, including the Observed Species (Sobs), Abundance-based Coverage Estimator (ACE), and Chao^1^ indices, were calculated. Differences among groups were assessed using the Kruskal–Wallis test. Beta diversity was assessed using unweighted UniFrac distances to quantify community compositional differences between samples. Principal Coordinate Analysis (PCoA) was performed on the resulting distance matrix to visually represent the separation of microbial communities among groups. The statistical significance of group clustering observed in the PCoA plot was tested using Permutational Multivariate Analysis of Variance (PERMANOVA) with 999 permutations. Differential Abundance and Functional Prediction: To identify bacterial taxa that were differentially abundant between specific groups, Linear Discriminant Analysis Effect Size (LEfSe) was performed, with a logarithmic LDA score threshold set to >2.0. The functional potential of the microbial communities was predicted from the 16S rRNA gene data using Phylogenetic Investigation of Communities by Reconstruction of Unobserved States 2 (PICRUSt2). Predicted functional abundances were annotated against the Kyoto Encyclopedia of Genes and Genomes (KEGG) database. Integration with Host Data: Associations between the relative abundance of microbial genera and host immune-inflammatory parameters were evaluated using Spearman’s rank correlation analysis. Results were visualized as a correlation heatmap.

### 2.10. Statistical Analysis

All quantitative data are presented as mean ± standard deviation (SD) unless otherwise specified. Prior to hypothesis testing, the normality of data distribution within each experimental group was assessed using the Shapiro–Wilk test. For comparisons involving more than two groups, continuous variables that met the normality assumption (e.g., cytokine levels, skin and ear thickness) were analyzed by one-way analysis of variance (ANOVA). When ANOVA indicated a significant overall effect, pairwise comparisons between groups were conducted using Tukey’s honestly significant difference (HSD) post hoc test. For comparisons of multiple treatment groups against a single control group (e.g., all interventions vs. the DNFB model group), Dunnett’s test was applied following ANOVA. Continuous data that violated the normality assumption, as well as ordinal data (e.g., histopathological scores), were analyzed using the non-parametric Kruskal–Wallis test. Significant results were followed by Dunn’s post hoc test with Bonferroni correction to control for multiple comparisons. For direct comparisons between two independent groups, either the independent-samples Student’s *t*-test (for normally distributed data) or the Mann–Whitney U test (for non-normally distributed data) was used. Categorical data, such as incidence rates (e.g., proportion of mice exhibiting redness or hair restoration), were analyzed using the Chi-square test or Fisher’s exact test, as appropriate. A two-tailed *p*-value < 0.05 was considered statistically significant. All statistical analyses were performed using SPSS software (version 25.0; IBM Corp., Armonk, NY, USA). Graphs were generated with GraphPad Prism (version Prism 9; GraphPad Software, San Diego, CA, USA). Histopathological sections were digitized and evaluated using CaseViewer software (version 2.9; 3D HISTECH, Budapest, Hungary).

## 3. Results

### 3.1. Effects on Skin Appearance in Mice with AD

**Figure 2:**
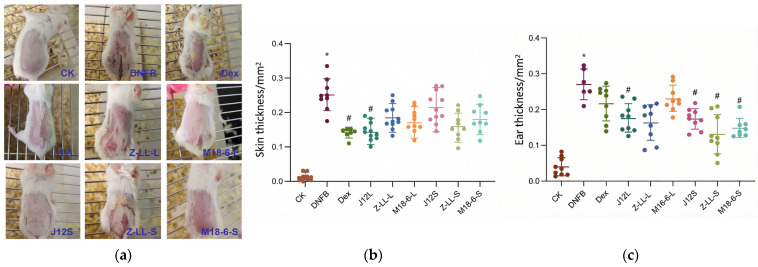
Macroscopic and biometric assessments of AD symptoms in mice following three probiotics interventions. (**a**) Representative dorsal skin photographs. Images were captured on day 49 (experimental endpoint) to illustrate the macroscopic appearance of skin lesions. (**b**) Measurement of dorsal skin thickening. Changes in dorsal skin thickness were measured using a digital caliper and are expressed as the percentage change from baseline (pre-modeling) values. Data are presented as mean ± SD (*n* = 12 mice per group). (**c**) Measurement of ear swelling. Ear thickness change was calculated as the difference between the right (sensitized) and left (non-sensitized) ears at the endpoint. Data are presented as mean ± SD (*n* = 6–9 mice per group). Statistical significance for (**b**,**c**) was determined by one-way ANOVA followed by Tukey’s post hoc test for multiple comparisons. * *p* < 0.05, vs. the CK group; # *p* < 0.05, vs. the DNFB group.

After DNFB modeling, the mice in the experimental groups, except for the CK group, displayed pronounced itching. The application of DNFB disrupted the skin barrier of the mice, leading to marked redness, swelling, and dryness on their dorsal skin lesions. However, following the oral administration of *B. animalis* J12 live cells and the topical application of J12 supernatant, the redness, swelling, and dryness of the back skin were significantly alleviated (Figure 2a). Notably, J12S increased hair restoration to 50% and reduced the redness and swelling rate to 66.7% significantly, respectively, compared to the DNFB group, which had rates of 8% and 100%. Compared to the CK group, DNFB induction led to a significant increase in the thickness of the dorsal skin and ears of the mice. However, in the J12L group, the changes in skin thickness and ear thickness were significantly reduced compared to the DNFB group (Figure 2b,c). This indicates that treatment with *B. animalis* J12 inhibits skin swelling caused by DNFB. *L. plantarum* Zhang-LL and *L. salivarius* M18-6 also demonstrated positive intervention effects on ear thickness. These findings suggest that *B. animalis* J12, as well as *L. plantarum* Zhang-LL and *L. salivarius* M18-6, have the potential to alleviate symptoms of AD (Table 1).

### 3.2. Effects on Skin Barrier in Mice with AD

**Figure 3:**
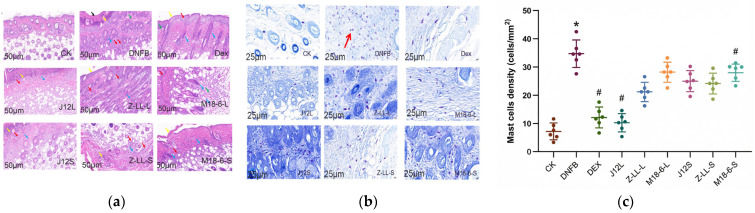
Histopathological evaluation of skin lesions and mast cell infiltration in AD mice treated with three probiotics. Dorsal skin tissue sections were stained and analyzed at the experimental endpoint. (**a**) Representative hematoxylin and eosin (H&E) stained sections. Images show the skin morphology of each group. Pathological features in the DNFB model group are indicated by arrows: blue arrow, connective tissue hyperplasia; red arrow, lymphocytic infiltration; yellow arrow, epidermal acanthosis (moderate thickening); black arrow: epidermal hyperkeratosis/parakeratosis; green arrow: eosinophil/ neutrophil infiltration. Scale bar = 50 µm. (**b**) Representative toluidine blue stained sections for mast cell identification. Mast cells are indicated by purple metachromatic granules (red arrow in DNFB group). Scale bar = 25 µm. (**c**) Quantitative analysis of mast cell density. The number of mast cells was counted in three random high-power fields (HPF, 400× magnification) per section and expressed as cells per mm^2^. Data are presented as mean ± SD (n = 6 mice per group). Statistical significance was determined by one-way ANOVA followed by Tukey‘s post hoc test. * *p* < 0.05 vs. the CK group; # *p* < 0.05 vs. the DNFB group.

The results of hematoxylin–eosin staining of pathological sections revealed that the epidermis structure in the CK group was intact, with normal morphology and closely arranged squamous epithelial cells. The dermis showed that a rich presence of collagen fibers and accessory organs such as hair follicles and sebaceous glands were visible. The subcutaneous muscle layer exhibited a complete and uniformly thick structure, with normal and regularly arranged muscle fibers, and no noticeable inflammation was observed. In contrast, the DNFB group displayed a large number of necrotic cell debris and epidermal thickening. Scattered infiltration of lymphocytes and neutrophils was observed in the dermis, along with a small amount of connective tissue proliferation at the dermis tip. However, no necrotic cell fragments were found in the J12S group (Figure 3a). Compared to the CK group, the DNFB group exhibited a significant increase in mast cell count, while the J12L group exhibited a significant decrease (Figure 3b,c).

### 3.3. Effects of B. animalis J12 on Immune System in Mice with AD

**Figure 4:**
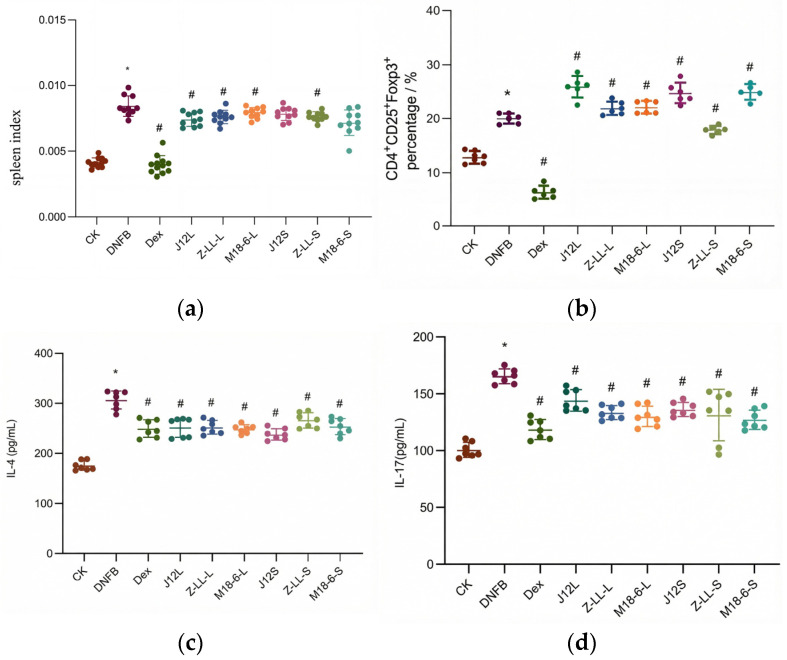
*B. animalis* J12 modulates systemic immune responses in AD mice. (**a**,**b**) Analysis of splenic regulatory T cells (Tregs). Splenocytes were isolated and analyzed by flow cytometry for CD4 + CD25 + Foxp3 + Tregs. (**a**) Representative flow cytometry plots gated on CD4 + T cells. (**b**) Quantitative summary of the percentage of Tregs among CD4 + T cells. (**c**,**d**) Measurement of serum inflammatory cytokines. Serum levels of (**c**) interleukin-4 (IL-4) and (**d**) interleukin-17 (IL-17) were quantified by ELISA. Data are mean ± SD (*n* = 7–12). (One-way ANOVA with Tukey‘s post hoc test). * *p* < 0.05 vs. the CK group; # *p* < 0.05 vs. the DNFB group.

As shown in Figure 4a,b, compared to the CK group, the DNFB group exhibited a significant increase in splenic regulatory T (Treg) cells (reflecting an adaptive immune feedback response). Both J12L and J12S treatments further up-regulated splenic Treg cells (4.54–4.64-fold vs. DNFB group) and suppressed pro-inflammatory cytokines. For serum cytokine levels (Figure 4c,d), the CK group had IL-4 (174.359 pg/mL) and IL-17 (100.791 pg/mL), while the DNFB group showed a significant elevation in IL-4 (306.661 pg/mL) and IL-17 (165.6 pg/mL). Both J12L and J12S significantly reduced these cytokine levels (vs. DNFB group).

### 3.4. Effects of B. animalis J12 on Intestinal Microorganisms in Mice with AD

Based on previous index analysis, *B. animalis* J12 exhibited superior efficacy in AD management; we thus further explored its potential effects on the gut microbiota–skin axis (a well-established link to AD pathogenesis). To assess α-diversity changes in AD mice’s gut flora, we quantified richness via the sobs, Ace, and Chao indices: relative to the CK group, the DNFB-induced AD model group showed significantly reduced sobs, Ace, and Chao indices (*p* < 0.05), while J12L intervention reversed this trend (*p* < 0.05). These results confirm that J12 live cells enhance gut microbial α-diversity, improving the richness of the intestinal microbiota in AD mice (Table 2).

**Figure 5:**
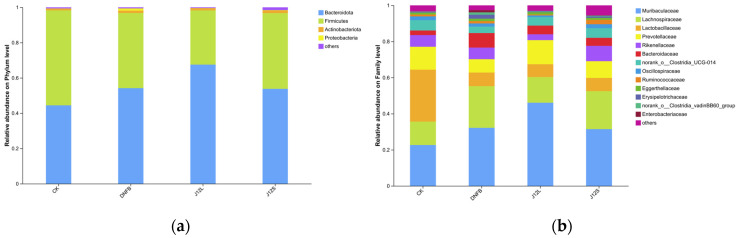

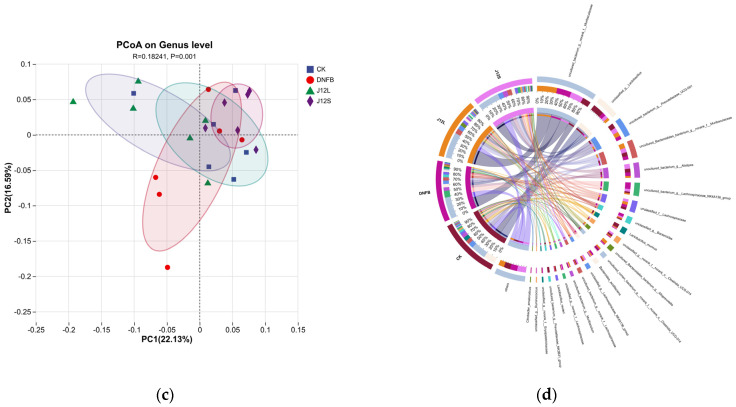
Gut microbiota modulation by *B. animalis* J12 in AD mice. Intestinal microbial composition (16S rRNA sequencing) at study endpoint: (**a**) Phylum-level relative abundance (stacked bars, dominant phyla labeled). (**b**) Family-level relative abundance (stacked bars, top 10 families). (**c**) Genus-level β-diversity (unweighted UniFrac PCoA; each point = 1 mouse; 95% confidence ellipses; PERMANOVA, *p* = 0.001). (**d**) Genus-level distribution (Circos plot; ribbons link top 10 genera (outer ring) to groups (inner ring); width = abundance). Data: mean (*n* = 6/group).

At the phylum level, the gut microbiota was dominated by *Firmicutes* and *Bacteroidetes* across all groups; while dominant phylum composition was consistent, relative abundances varied (Figure 5a). At the family level, the CK group was primarily composed of *Muribaculaceae*, *Lachnospiraceae*, *Lactobacillaceae*, *Prevotellaceae*, and *Rikenellaceae*. The DNFB group showed decreased *Lactobacillaceae* and *Prevotellaceae* abundances (with *Lactobacillaceae* dropping from 28.7% (CK) to 7.56%), while J12 intervention elevated *Prevotellaceae* but further reduced *Lactobacillaceae* (7.1% in J12L, 7.29% in J12S) (Figure 5b).

We next analyzed genus-level β-diversity via unweighted UniFrac PCoA: ANOSIM confirmed significant intergroup differences in community structure (*p* = 0.001; Figure 5c). Given the lack of significant dominant community shifts at the phylum/family level, a Circos plot was used to resolve genus-level variations (Figure 5d).

### 3.5. Species-Level Differential Abundance Analysis

**Figure 6:**
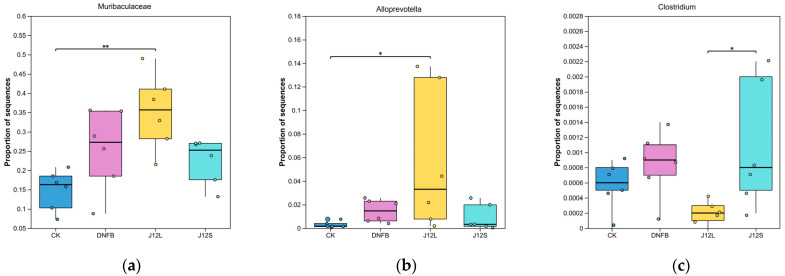

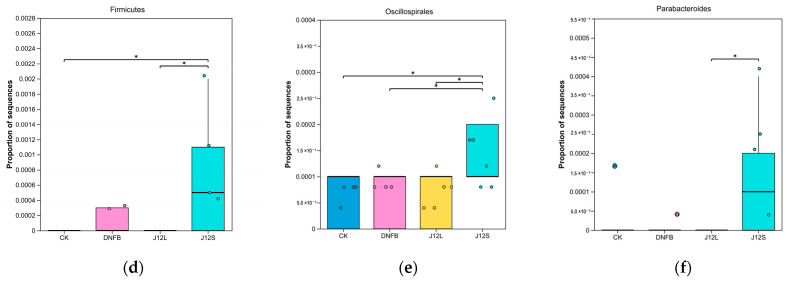
Species-level differential abundance of core gut microbial taxa. Boxplots depicting the relative abundance of six key taxa across four experimental groups (CK, DNFB, J12L, J12S): (**a**) *Muribaculaceae*, (**b**) *Alloprevotella*, (**c**) *Clostridium*, (**d**) *Firmicutes*, (**e**) *Oscillospirales*, and (**f**) *Parabacteroides*. Each dot represents an individual mouse sample; significance markers (derived from Kruskal–Wallis H tests followed by pairwise comparisons) indicate *p* < 0.05 (*) and *p* < 0.01 (**).

To characterize species-level microbial shifts associated with AD and J12 intervention, we performed nonparametric analysis for multi-group abundance differences followed by pairwise comparisons. Six species-level taxa demonstrated significant intergroup abundance variations; *Muribaculaceae* exhibited a highly significant difference (*p* < 0.01), with higher abundance in the DNFB group than the CK group (Figure 6a), and *Alloprevotella*, *Clostridium*, *Firmicutes*, *Oscillospirales*, and *Parabacteroides* (Figure 6b–f) all showed significant differences (*p* < 0.05), with *Alloprevotella* enriched in the J12L group and *Clostridium*, *Firmicutes*, *Oscillospirales*, and *Parabacteroides* elevated in the J12S group relative to the DNFB model group (with *Oscillospirales* also differing from CK/J12L). These data demonstrate that *B. animalis* J12 modulates distinct core gut taxa, with oral live cells and topical supernatant driving divergent regulatory patterns in AD mice.

**Figure 7:**
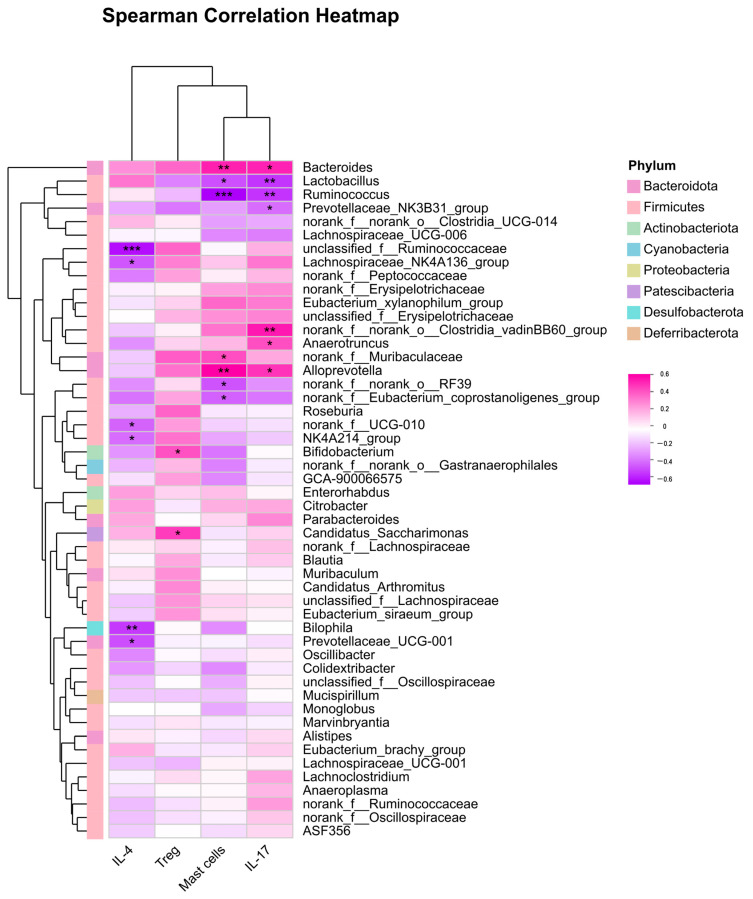
This Spearman correlation heatmap displays correlations between species (*Y*-axis) and environmental factors (*X*-axis), with R-values indicated by color gradients (right scale) and significance (*p* ≤ 0.05) marked as *p* ≤ 0.05 (*), 0.001 < *p* ≤ 0.01 (**), *p* ≤ 0.001 (***). Hierarchical clustering (left) groups species by similar correlation patterns.

The Spearman correlation coefficient in Figure 7 was utilized to analyze the correlation between immune inflammatory indicators (IL-4, IL-17, Treg cells, mast cells) and the intestinal flora. The heatmap analysis revealed the following correlations: first, mast cells demonstrated a significant negative correlation with *Lachnospiraceae*, *Colidextribacter*, *Ruminococcus*, *Oscillospiraceae*, *Oscillibacter*, *Mucispirillum*, and *Butyricicoccus*, while showing a significant positive correlation with *Muribaculaceae* and *Alloprevotella*. Secondly, IL-17 displayed a significant negative correlation with *Lactobacillus*, *Prevotellaceae*, *Lachnospiraceae*, *Oscillibacter*, and *Mucispirillum*, and a significant positive correlation with *Muribaculaceae* and *Alloprevotella*. Moreover, *Lactobacillus* exhibited a significant negative correlation with mast cells and IL-17, while *Lachnospiraceae*-UCG-001 was significantly negatively correlated with Treg cells. *Bifidobacterium* was positively correlated with Treg cells. Additionally, *Ruminococcus* showed a significant negative correlation with mast cells and IL-17.

## 4. Discussion

### 4.1. Both Topical and Oral J12 Interventions Effectively Alleviate Experimental AD

This study provides clear evidence that *B. animalis* J12, administered either orally as live cells (J12L) or topically as a fermented supernatant (J12S), effectively mitigates AD in mice. Both interventions significantly improved key clinical and histopathological hallmarks of AD, including skin erythema, swelling, epidermal hyperplasia, and mast cell infiltration (Figure 2; Table 1). Notably, topical J12S demonstrated efficacy comparable to oral J12L, presenting a novel, non-invasive application route for probiotic-based therapy. Specifically, oral J12L significantly altered the composition of the mouse gut microbiota, while topical J12S had no obvious impact on the gut microbiota. This administration-route-dependent difference strongly demonstrates that the mechanism by which J12 exerts its AD-improving effect is pathway-specific, rather than originating from non-specific factors such as moisturization or pH changes that may be associated with topical interventions (Figure 5, Figure 6 and Figure 7). Further analysis at the biomarker level showed that J12 intervention reduced mast cell infiltration and significantly downregulated the levels of inflammatory cytokines (e.g., IL-4, IL-17) in the skin tissues of AD mice (Figure 4). These specific biological changes further rule out the dominant role of non-specific mechanisms such as “simple barrier moisturization” and confirm the specificity of J12 in improving AD by regulating immune-inflammatory responses.

### 4.2. Modulation of Skin Barrier Integrity and Local Inflammatory Response

The therapeutic benefits of J12 are closely linked to its modulation of local skin inflammation and maintenance of skin barrier function. Cell necrosis promotes inflammatory responses, which are critical in skin and intestinal inflammation [23,24,25]. Mast cells, widely distributed in the skin, play a key role in antigen-mediated allergic reactions and thus are pivotal in allergic skin diseases [26]. As a common feature of AD, pruritus is triggered by mast cell degranulation-released prostaglandins, inflammatory cytokines, and chemokines that infiltrate tissues. We therefore counted mast cells in the dorsal skin of mice in each group using toluidine blue staining. Kim et al. reported that *Kazachstania turicensis* CAU Y1706 significantly reduced mast cell counts in AD mice [27,28]; consistently, both J12L and J12S decreased mast cell infiltration (Figure 2)—a key effector cell in allergic pruritus—likely contributing to reduced epidermal thickness and cutaneous inflammation. However, their mechanisms differ: oral J12L uniquely restored gut microbiota alpha diversity and more profoundly reduced mast cell infiltration, while topical J12S exerted direct local skin barrier protection without altering gut ecology [29,30].

### 4.3. Restoration of Systemic Immune Homeostasis

Beyond local effects, J12 induced profound systemic immunomodulation. A key finding was the significant increase in splenic regulatory T cells (Tregs) following both J12L and J12S treatment (Figure 4a,b). Tregs are pivotal for maintaining immune tolerance and suppressing aberrant activation [31,32,33], and their expansion aligns with established probiotic mechanisms in alleviating allergic inflammation [34,35]. Both J12L and J12S, as well as *L. plantarum* Zhang-LL and *L. salivarius* M18-6, significantly decreased IL-4 and IL-17 levels compared to the DNFB group, indicating regulation of Th2/Th17 cytokines—consistent with prior findings that intragastric *B. longum* reduces inflammatory factors [36]. Both J12 treatments lowered serum IL-4 (Th2) and IL-17 (Th17) (Figure 4c,d), correcting AD’s immune imbalance. While the DNFB group showed compensatory Treg elevation, J12′s further Treg amplification combined with cytokine suppression reflects effective immune regulation rather than mere quantitative change.

### 4.4. Oral J12L Operates via Gut Microbiota Colonization and Metabolic Regulation

Oral J12L’s systemic effects are mediated through the gut–skin axis [37]. 16S rRNA sequencing confirmed J12L colonizes the intestine, restoring AD-diminished gut microbiota alpha diversity (Sobs, ACE, Chao^1^ indices) and reshaping composition (e.g., elevated *Prevotellaceae*, modulated *Lactobacillaceae*)—taxa associated with anti-inflammatory responses and gut barrier integrity (Table 1; Figure 5). At the family level, J12L elevated *Prevotellaceae* abundance, which was reduced in the DNFB group, while maintaining *Lactobacillaceae* at a low level similar to the model group. This specific regulation pattern differs from other probiotics like *L. plantarum* NCIMB8826, which increases *Lactobacillaceae* abundance to exert anti-AD effects [14], indicating J12L’s unique mechanism of gut microbiota modulation. At the species level, J12L significantly enriched *Alloprevotella*, negatively correlated with IL-4 and IL-17, suggesting direct involvement in suppressing pro-inflammatory responses. Our prior study demonstrated that both live and heat-killed J12 exert mucosal protective effects [18], indicating efficacy involves viable colonization and bioactive components from live/dead bacteria [38]. Key mediators are microbial metabolites like SCFAs (e.g., butyrate), which promote Treg differentiation via epigenetic mechanisms and receptor signaling (e.g., GPR41/GPR43) [39], modulate inflammatory responses via G-protein-coupled receptors [40], and mediate gut–skin crosstalk by influencing distant immune responses through regulating epithelial integrity and immune homeostasis [41,42]. Spearman correlation analysis further confirmed that J12L-modulated gut taxa are closely linked to AD-related immune parameters: Lactobacillus was negatively correlated with mast cells and IL-17, *Lachnospiraceae* and *Ruminococcus* showed negative correlations with mast cell infiltration, and *Bifidobacterium* was positively correlated with Treg cells (Figure 7). These correlations support the mechanism axis oral J12L → gut colonization and microbial reshaping → SCFA production → systemic Treg induction and Th2/Th17 suppression. Collectively, J12′s colonization capacity and dual efficacy of live and heat-killed forms enhance its versatility in regulating gut microbiota and the gut–skin axis to alleviate AD pathology.

### 4.5. Local Immunomodulation by Topical J12S and Microbiota-Immunity Functional Links

Distinct from J12L, topical J12S exerts therapeutic effects via localized immunomodulation, while Spearman correlation analysis clarifies J12-modulated gut microbiota-AD immune crosstalk (Figure 7). Unlike J12L, J12S had no significant impact on gut microbial alpha diversity (Sobs, ACE, Chao^1^ indices showed no statistical difference compared to the DNFB group; Table 1) and only slightly upregulated the abundance of taxa such as *Clostridium*, *Oscillospirales*, and *Parabacteroides* at the species level (Figure 6). These minor changes did not alter the overall gut microbiota structure (Figure 5c), confirming J12S’s therapeutic effects are independent of gut flora modulation. J12S contains bioactive molecules (e.g., SCFAs, organic acids, peptides, extracellular vesicles) derived from *B. animalis* J12 metabolism, which interact with skin-resident immune cells and sensory neurons, improving barrier integrity and reducing mast cell infiltration. Local inflammation attenuation propagates systemic anti-inflammatory signals, explaining elevated splenic Tregs and reduced serum pro-inflammatory cytokines in J12S-treated mice [43]. Concurrently, correlation analysis revealed mast cells negatively correlated with *Lachnospiraceae*, *Colidextribacter*, *Ruminococcus*, *Oscillospiraceae*, *Oscillibacter*, *Mucispirillum*, and *Butyricicoccus*, and positively with *Muribaculaceae* and *Alloprevotella*; IL-17 showed analogous correlations plus negative associations with *Lactobacillus* and *Prevotellaceae*; *Lactobacillus* negatively correlated with mast cells and IL-17 (aligning with evidence of *lactobacilli* modulating inflammatory cytokines and mast cell activation); and *Bifidobacterium* positively correlated with Tregs. These links, paired with J12S’s local effects, underscore J12′s dual complementary pathways—direct cutaneous regulation by J12S bioactives and indirect gut microbiota-immune crosstalk—both restoring immune homeostasis in AD mice.

## 5. Conclusions and Limitations

This study shows that J12L and J12S both effectively alleviate DNFB-induced atopic dermatitis in mice, significantly improving histopathological features (skin lesions, ear swelling, pro-inflammatory cytokines IL-4/IL-17, splenic Treg proportion). Their mechanisms differ: J12S modulates the local skin immune microenvironment, exerting direct anti-inflammatory effects and skin barrier protection as a non-invasive option. J12L, besides affecting systemic immunomodulation, effectively colonizes the intestine, restores gut microbiota alpha diversity (Sobs, ACE, Chao^1^) and alters taxa abundance (e.g., *Prevotellaceae*, *Lactobacillaceae*), acting via the gut–skin axis (unique to J12L). Notably, both live and heat-killed J12 are bioactive, with J12S showing efficacy comparable to J12L. Their complementary mechanisms (systemic gut–skin axis regulation vs. local anti-inflammation) highlight J12′s potential in diversified AD treatment, supporting the development of stable, non-viable probiotic-derived topical formulations for clinical translation.

Although this study provides valuable evidence for J12′s application in AD management, it has limitations. Experimental design: To adhere to the “Reduction” principle of the 3Rs in animal research, healthy J12-only control groups and non-fermented supernatant groups were omitted; though this does not affect conclusions on therapeutic efficacy versus the AD model group, it limits mechanistic dissection. Uncharacterized bioactive components: The specific bioactive components in J12S responsible for the observed anti-inflammatory and skin barrier-protective effects have not been identified and characterized, limiting a deeper understanding of its mode of action. Future studies should focus on isolating and identifying the bioactive components in J12S and investigating the role of prebiotic substrates in enhancing J12 efficacy.

## Figures and Tables

**Table 1:** Visual effects of treatment on skin.

Group	Redness Swelling Rate, n/N (%)	Hair Restoration, n/N (%)
CK	0/12 (0.0)	12/12 (100.0)
DNFB	12/12 (100.0) *	1/12 (8.3) *
Dex	12/12 (100.0) *	8/12 (66.7) *#
J12L	11/12 (91.7) *	2/12 (16.7) *
Z-LL-L	12/12 (100.0) *	1/12 (8.3) *
M18-6-L	10/12 (83.3) *	4/12 (33.3) *
J12S	8/12 (66.7) *#	6/12 (50.0) *#
Z-LL-S	12/12 (100.0) *	0/12 (0.0) *
M18-6-S	12/12 (100.0) *	1/12 (8.3) *

Data are presented as number of mice exhibiting the symptom over total mice in the group (percentage). Significance was determined by Chi-square test. * *p* < 0.05 vs. the CK group; # *p* < 0.05 vs. the DNFB group.

**Table 2:** Alpha diversity indices of the gut bacterial community in different experimental groups.

Group	Observed ASVs (Sobs)	ACE	Chao^1^ Index
CK	290.16 ± 36.96	327.65 ± 30.93	330.14 ± 33.57
DNFB	235.5 ± 34.75 *	262.45 ± 39.76 *	262.84 ± 37.74 *
J12L	289.17 ± 9.62 ^#^	319.27 ± 16.13 ^#^	319.37 ± 18.97 ^#^
J12S	247.67 ± 45.53	290.97 ± 30.13	287.10 ± 47.06

Data are presented as mean ± standard deviation (SD). Alpha diversity indices were calculated from 16S rRNA gene sequencing data of intestinal contents. Abbreviations: Sobs, observed amplicon sequence variants; ACE, abundance-based coverage estimator; Chao, Chao^1^ richness estimator. Statistical significance was determined by Kruskal–Wallis test followed by Dunn‘s post hoc test. * *p* < 0.05, vs. the CK group; # *p* < 0.05, vs. the DNFB group.

## Data Availability

16S rRNA amplicon sequencing data are available at the Sequence Read Archive (Bioproject ID: PRJNA1161804) https://www.ncbi.nlm.nih.gov/sra (accessed on 10 March 2024). The data that support the findings of this study are available from the corresponding author upon reasonable request.
